# A novel prognostic signature and immune microenvironment characteristics associated with disulfidptosis in papillary thyroid carcinoma based on single-cell RNA sequencing

**DOI:** 10.3389/fcell.2023.1308352

**Published:** 2023-11-14

**Authors:** Zhenyu Liao, Ye Cheng, Huiru Zhang, Xing Jin, Hanxing Sun, Yue Wang, Jiqi Yan

**Affiliations:** ^1^ Department of General Surgery, Ruijin Hospital, Shanghai Jiao Tong University School of Medicine, Shanghai, China; ^2^ Institutes of Biomedical Sciences and Children’s Hospital, Fudan University, Shanghai, China; ^3^ Shanghai Cancer Centre, Fudan University, Shanghai, China; ^4^ Department of Thoracic Surgery, Zhongshan Hospital, Fudan University, Shanghai, China

**Keywords:** papillary thyroid carcinoma, prognostic signature, disulfidptosis, immune infiltration, endothelial cells

## Abstract

**Background:** Disulfidptosis is a newly discovered form of regulated cell death. The research on disulfidptosis and tumor progression remains unclear. Our research aims to explore the relationship between disulfidptosis-related genes (DRGs) and the clinical outcomes of papillary thyroid carcinoma (PTC), and its interaction on the tumor microenvironment.

**Methods:** The single-cell RNA seq data of PTC was collected from GEO dataset GSE191288. We illustrated the expression patterns of disulfidptosis-related genes in different cellular components in thyroid cancer. LASSO analyses were performed to construct a disulfidptosis associated risk model in TCGA-THCA database. GO and KEGG analyses were used for functional analyses. CIBERSORT and ESTIMATE algorithm helped with the immune infiltration estimation. qRT‒PCR and flow cytometry was performed to validate the hub gene expression and immune infiltration in clinical samples.

**Results:** We clustered PTC scRNA seq data into 8 annotated cell types. With further DRGs based scoring analyses, we found endothelial cells exhibited the most relationship with disulfidptosis. A 4-gene risk model was established based on the expression pattern of DRGs related endothelial cell subset. The risk model showed good independent prognostic value in both training and validation dataset. Functional enrichment and genomic feature analysis exhibited the significant correlation between tumor immune infiltration and the signature. The results of flow cytometry and immune infiltration estimation showed the higher risk scores was related to immuno-suppressive tumor microenvironment in PTC.

**Conclusion:** Our study exhibited the role of disulfidptosis based signature in the regulation of tumor immune microenvironment and the survival of PTC patients. A 4-gene prognostic signature (including SNAI1, STC1, PKHD1L1 and ANKRD37) was built on the basis of disulfidptosis related endothelial cells. The significance of clinical outcome and immune infiltration pattern was validated robustly.

## 1 Introduction

Thyroid cancer is the most common endocrine malignancy (Chen et al., 2016). Papillary thyroid carcinoma (PTC) account for almost 80% of thyroid cancer cases, and its incidence is rapidly increasing in worldwide (Haugen et al., 2016; Siegel et al., 2022). Although most thyroid cancers have a good prognosis and can be treated surgically, there is still a lack of standard treatment for those highly aggressive and poorly differentiated tumors, which are prone to progression to advanced tumors (Giannini et al., 2019; Ruiz et al., 2023). There is a rather part of patients not sensitive to radioactive iodine (RAI) ablation and thyroid stimulating hormone (TSH) suppression treatment (Tiedje and Fagin, 2020). Thus, it is urgent to find new molecular targeted therapies and immunotherapies for these aggressive tumors. Compared to other solid tumors, the tumor ecosystem of PTC remains poorly characterized and new insights are needed to explore the progression of papillary thyroid carcinoma (Pu et al., 2021).

Disulfidptosis is a newly discovered type of regulated cell death (RCD), different from apoptosis and ferroptosis, this procedure cannot be mitigated by previous inhibitors of cell death (Liu et al., 2023). It is found that under glucose starvation situation the expression of solute carrier family 7 member 11 can induce the abnormal accumulation of cystine and the other disulfide (Liu et al., 2021). The formation of these disulfide bonds between actin cytoskeletal results in the collapse of the cytoskeleton structure and eventually cell death. Further, the treatment of glucose transporter (GLUT) inhibitors can trigger disulfidptosis which indicates that the inducement of disulfidptosis might be a promising therapeutic strategy (Zheng et al., 2023). It is also reported that the disulfidptosis procedure is closely related to the regulation of immune response in multiple tumor microenvironment (Qi et al., 2023; Zhao et al., 2023).

Since the research on disulfidptosis in cancer progression is still in its initial stage, this study comprehensively collected the reported disulfidptosis-related genes (DRGs) and further combined papillary thyroid carcinoma scRNA-seq dataset and bulk RNA-seq database from the cancer genome atlas (TCGA) to explore the key cell cluster associated with disulfidptosis. The hub genes were selected to construct a risk model based on DRGs under LASSO Cox regression algorithm. The downstream analysis proclaims the function of the risk model in clinical prognosis, genomic features, functional enrichment and immune microenvironment investigation. Our comprehensive study provides new sight into disulfidptosis related tumor heterogeneity and therapeutic targets in thyroid cancer.

## 2 Materials and methods

### 2.1 Data collection

The PTC single-cell RNA sequencing dataset (GSE191288) was obtained from the Gene Expression Omnibus (GEO) repository. The mRNA sequencing data of patients with thyroid cancer were downloaded from The Cancer Genome Atlas (TCGA) (https://portal.gdc.cancer. gov/projects/TCGA-THCA). The pathological data was screened in which only papillary thyroid carcinoma was retained. These data were used for the analysis of cell clustering and the establishment and validation of a DRGs based prognosis model.

### 2.2 Patient samples

All postoperative thyroid specimens were collected from Ruijin Hospital, Shanghai Jiao Tong University School of Medicine. Specimens were collected in accordance with institutional protocols and informed consent were obtained. For flow cytometry, fragments of fresh tumor tissue specimens were digested by Liberase TL (Roche Diagnostics) and DNase I (Roche Diagnostics) for 30 min. Single cells were filtered through 70 μm cell strainers and re-suspended in Percoll (40%, GEHealthcare) for gradient centrifugation, as previously described (Liao et al., 2023).

### 2.3 Flow cytometry and antibodies

Dead cells were first excluded by using Fixable Viability Dye eFluor780 (eBioscience, San Diego, CA). Intracytoplasmic staining was performed under the instructions of Fix/Perm Kit (BD Biosciences). The following antibodies were used for the human specimens: anti-CD45 (HI30), anti-CD68 (Y1/82A), anti-CD3 (OKT3), anti-CD8 (SK1) and anti-CD4 (SK3). All antibodies were purchased from BioLegend (San Diego, CA). Flow cytometric analysis was performed on an LSRFortessa system (BD Biosciences, San Jose, CA). All the FACS plots were analyzed and plotted by FlowJo V10.8.1.

### 2.4 Primers and quantitative real-time PCR (qRT‒PCR)

Primer for SNAI1: 5′- TCG​GAA​GCC​TAA​CTA​CAG​CGA -3’ (forward), 5′- AGATGAGCATTGG CAGCGAG-3’ (reverse). Primer for STC1: 5′- GTG​GCG​GCT​CAA​AAC​TCA​G -3’ (forward), 5′- GTGG AGCACCTCCGAATGG -3’ (reverse). Total RNA of cells was extracted using TRIzol reagent (Invitrogen, USA). cDNA was obtained by reverse transcription using a Vazyme HiScript III RT SuperMix for qPCR reagent kit. The qRT‒PCR was performed on an ABI 7900HT Real-Time PCR system (Applied Biosystems, USA).

### 2.5 The process of scRNA dataset and cell annotation

The routine process followed the Seurat v3 guidelines, which included the cell QC procedure, normalization and PCA dimensional reduction. The harmony R package was used for batch effect removing (Korsunsky et al., 2019). After PCA dimensional reduction, different cell clusters were labeled with the first 20 PCs and a resolution value of 0.4. Marker genes manually to match the cell annotation in the CellMarker database (http://biocc.hrbmu.edu.cn/CellMarker/) with SingleR and scType R package (Aran et al., 2019; Choi et al., 2020). The marker genes of each subset clusters were conducted by FindAllMarkers with the default parameters.

### 2.6 Cell cluster score based on disulfidptosis-related genes (DRGs)

Through reviewing the literatures related to disulfidptosis, we collected a disulfidptosis-related gene set of 107 genes (Supplementary Table S1). To evaluate the correlation between different cell clusters and disulfidptosis procedure, an assessment was conducted by the DRGs gene set based scoring algorithm. Six independent scoring algorithms were used, including AUCell, singscore, UCell, ssGSEA, JASMINE and viper scores. These scores were normalized and visualized by boxchart.

### 2.7 Cell-cell communication networks

We applied the Cellchat19 (v1.4.0) R package for a systematic analysis of cell-cell communication networks. Cellchat have a database involving interactions among ligands, receptors and their cofactors, identifiying communications between 2 cell groups mediated by these signaling genes, and associating each interaction with a probability value, so as to significantly identify the interaction probability under the randomly permutes statistic test (Jin et al., 2021). The visualization of these cell-cell interactions was also performed by Cellchat19.

### 2.8 LASSO cox regression analysis

To establish a prognostic signature, we employed the LASSO penalized Cox proportional hazards regression technique through the glmnet R package. The optimal lambda was determined according to the result of a maximum cross-validation likelihood calculation. The caret R package was applied to build the classification of the TCGA-THCA cohort and assess machine learning classifiers for the classification task. The Kaplan–Meier survival curves of both data set were plotted using survival R package.

### 2.9 Functional enrichment analysis

Gene Ontology (GO) and Kyoto Encyclopedia of Genes and Genomes (KEGG) pathway analyses were performed to evaluate the functional enrichment of the DEGs. GO analysis indicated the possible role of differentially expressed target genes in the cellular component (CC), molecular functions (MFs), and biological processes (BPs). KEGG pathway analysis revealed the signaling pathways involved in the regulation of cell function by these genes.

### 2.10 Immune landscape estimation and genomic analysis

The immune cell infiltration estimation of TCGA was based on CIBERSORT algorithm, and the stromal and immune microenvironment estimation was done by ESTIMATE algorithm (Yoshihara et al., 2013; Newman et al., 2015). The maftools R package was employed to analyze the gene mutation pattern, and tumor mutational burden (TMB) was calculated as the number of somatic base substitutions or indels per megabase (Mb) of the coding region target territory of the test, as previously described (Jin et al., 2022).

### 2.11 Statistical analysis

The statistical and bioinformatic analysis was conducted by R 4.2.0. The Log-rank survival analysis and Cox proportional hazards regression were performed using R package survival and survminer. The establishment and validation of the nomogram were performed and plotted using rms and Hmisc R package. The statistical analysis was performed via unpaired Student’s t-test analysis or Wilcoxon signed rank test unless specified. All *p* values were two-sided, and *p* < 0.05 were considered statistical significance (∗*p*-value <0.05, ∗∗*p*-value < 0.01, ∗∗∗*p*-value <0.001).

## 3 Results

### 3.1 Single-cell sequencing analysis of PTC and normal thyroid samples

**FIGURE 1:**
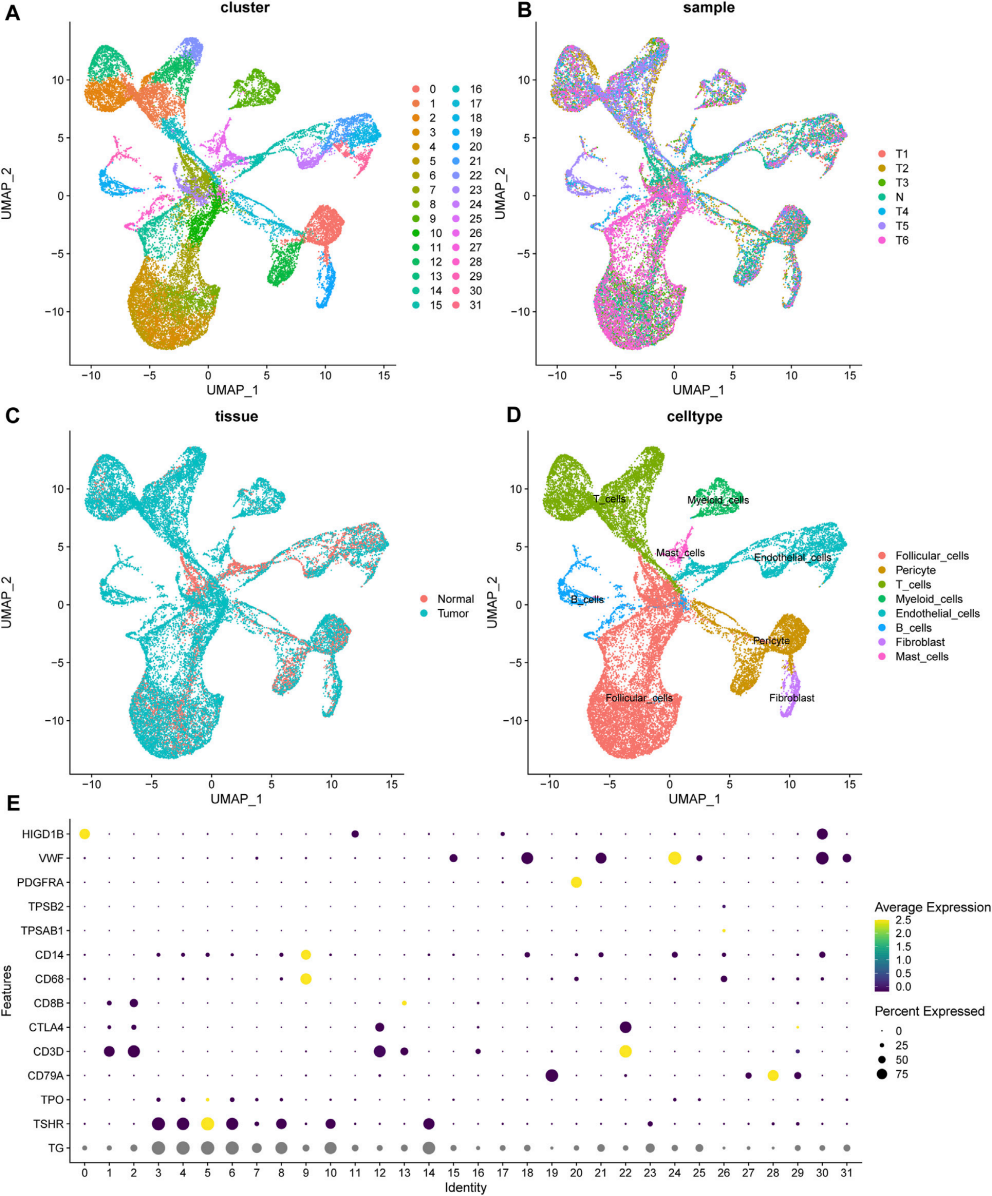
The landscape of scRNA seq of thyroid samples. **(A)** The unified manifold approximation and projection (UMAP) of 32 cell clusters. **(B, C)** The cells from 1 normal and 6 tumor samples. **(D)** The cell annotation of the clusters. **(E)** The universal marker gene used for cell annotation. TG, TSHR, TPO for follicular cells; CD79A for B cells; CD3D, CTLA4, CD8B for T cells; TPSAB1 for mast cells; PDGFRA for fibroblasts; VWF for endothelial cells; HIGD1B for pericytes.

With the help of single-cell RNA sequencing, we have a better understanding of the cellular components of thyroid cancer. The sequencing of 6 tumor samples from 3 patients and 1 normal sample were obtained from dataset GSE191288 (Wang et al., 2022). After normalization and dimensionality reduction through UMAP algorithm, cells were segregated into 32 clusters (Figure 1A). Using the FindAllMarkers function, the signature genes of each cluster was defined. The normal and tumor sample origin of these cells was also visualized (Figures 1B, C). With the analysis of the representing gene markers of these clusters, the final cell annotation was completed via the Cellmarker database. 32 clusters were categorized into 8 cell types, including follicular cells, pericytes, T cells, Myeloid cells, endothelial cells, B cells, fibroblasts and mast cells (Figure 1D). The universal marker genes which classified the main cell types were shown in the dotplot (Figure 1E).

### 3.2 Endothelial cells show closest relationship to DRGs in PTC

**FIGURE 2:**
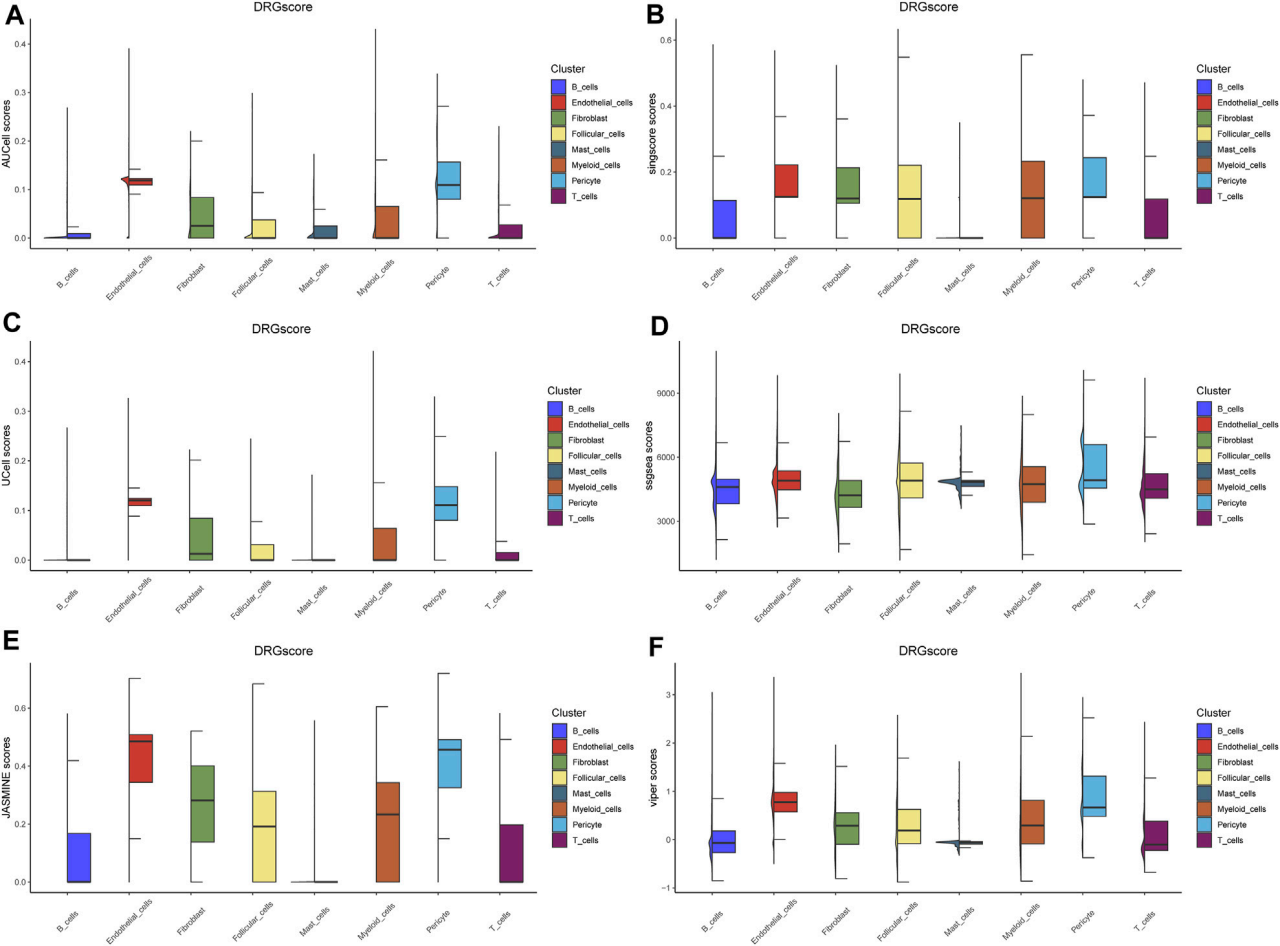
Cell cluster scoring based on DRGs. **(A–F)** The boxplot of 8 cell clusters under AUCell, singscore, UCell, ssGSEA, JASMINE and viper DRGs based scores.

Through reviewing the reported articles, we collected a 107-gene set of disulfidptosis-related genes (DRGs). In order to analysis the correlation between the different cell types in PTC and DRGs expression module, we applied a series of scoring algorithms, including AUCell, singscore, UCell, ssGSEA, JASMINE and viper scores (Figures 2A–F). Eight different cell types annotated in scRNA data were scored by 6 independent scoring algorithms. Among the results, we found that endothelial cells and pericytes showed the best correlation with DRGs. With further cell-cell interaction network analysis endothelial cells also showed a close connection to the tumoral cell entities (Supplementary Figure S1). It is also reported that these DRGs were most abundantly enriched in endothelial cells in lung cancer (Ni et al., 2023). The critical role of endothelial cells with the ability of sprouting angiogenesis in PTC tumor progression has already been issued (Winnik et al., 2009; Pu et al., 2021). Thus, we targeted on the endothelial cells and DRGs in the following study.

### 3.3 The expression pattern of DRGs related endothelial cell subsets

**FIGURE 3:**
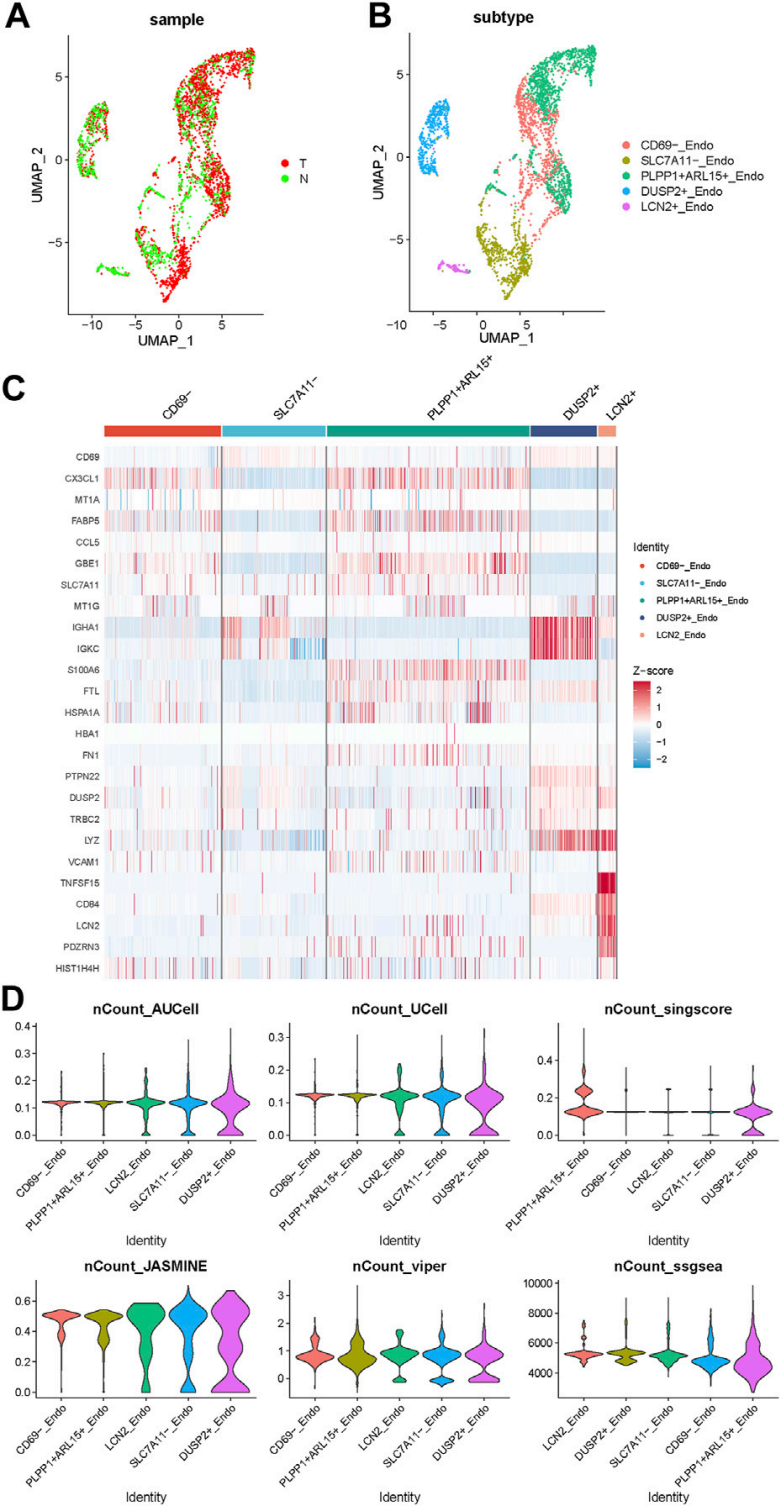
The expression pattern of endothelial cell subsets in PTC. **(A)** The endothelial cells from normal and tumor samples. **(B)** The UMAP of 5 subsets of endothelial cells. **(C)** The gene expression pattern of 5 endothelial cell subsets. **(D)** The violinplot of 5 subsets under AUCell, singscore, UCell, ssGSEA, JASMINE and viper DRGs based scores.

To further explore the role of endothelial cells in disulfidptosis related procedures, we used PCA algorithm to distinguish the different subsets of endothelial cells (ECs) in PTC (Figures 3A, B). ECs were categorized into 5 different subsets: CD69^−^ subset, SLC7A11^-^ subset, PLPP1^+^ ARL15^+^ subset, LCN2^+^ subset and DUSP2^+^ subset according their representative gene expression patterns (Figure 3C). The AUCell, singscore, UCell, ssGSEA, JASMINE and viper scores of DRGs were applied in these 5 subsets, the result showed that DUSP2^+^ subset was the best correlated endothelial cell subset with the high expression of IGHA1, IGKC and LYZ, while CD69-subset was the least relevant subtype with the high expression of CX3CL1 and FABP5. The differentially expressed genes (DEGs) between these two subsets might indicate a special expression signature as a bridge connecting DRGs and endothelial cells in PTC.

### 3.4 A prognostic signature based on DRGs related endothelial cells

**FIGURE 4:**
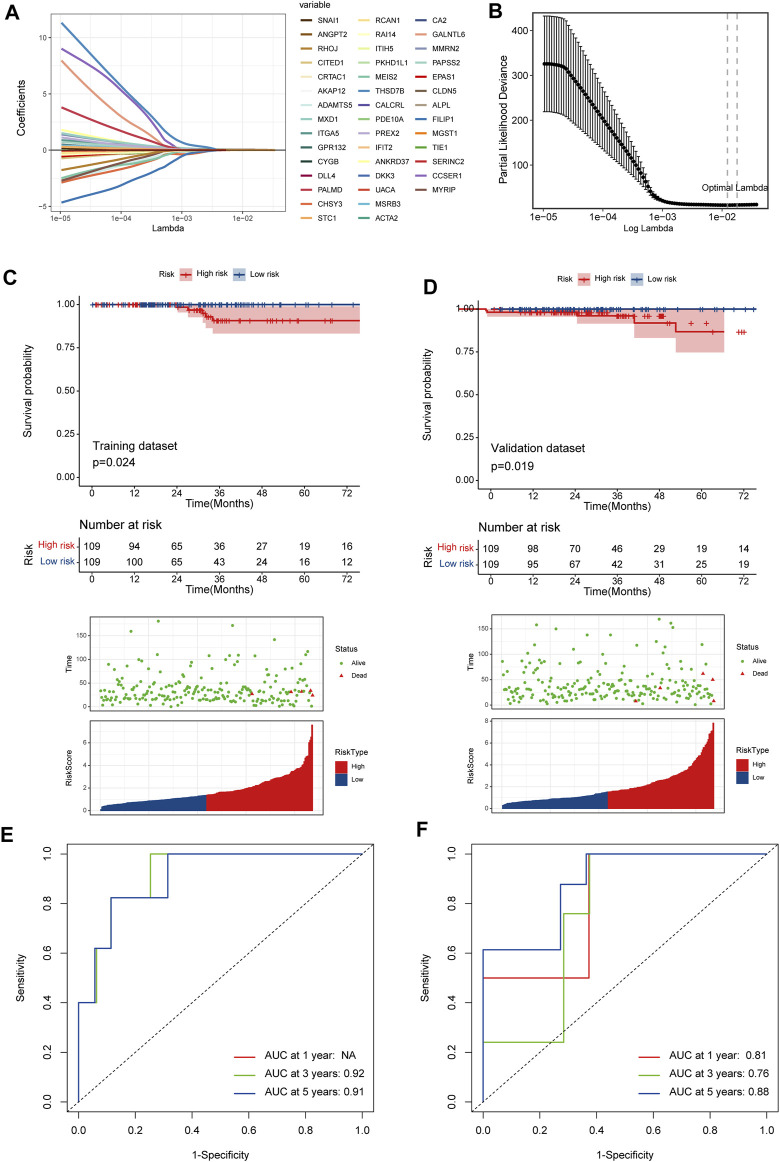
A prognostic signature based on DRGs related endothelial cells. **(A)** The LASSO coefficient profile plot shows the correlation between the deviance and log(λ). **(B)** The partial likelihood of deviance for the LASSO Cox regression analysis. **(C)** The survival plot of the high-risk group and low-risk group in the training dataset. **(D)** The survival plot of the high-risk group and low-risk group in the validation dataset. **(E, F)** The ROC curve plots of the prognostic model in training and validation dataset.

**FIGURE 5:**
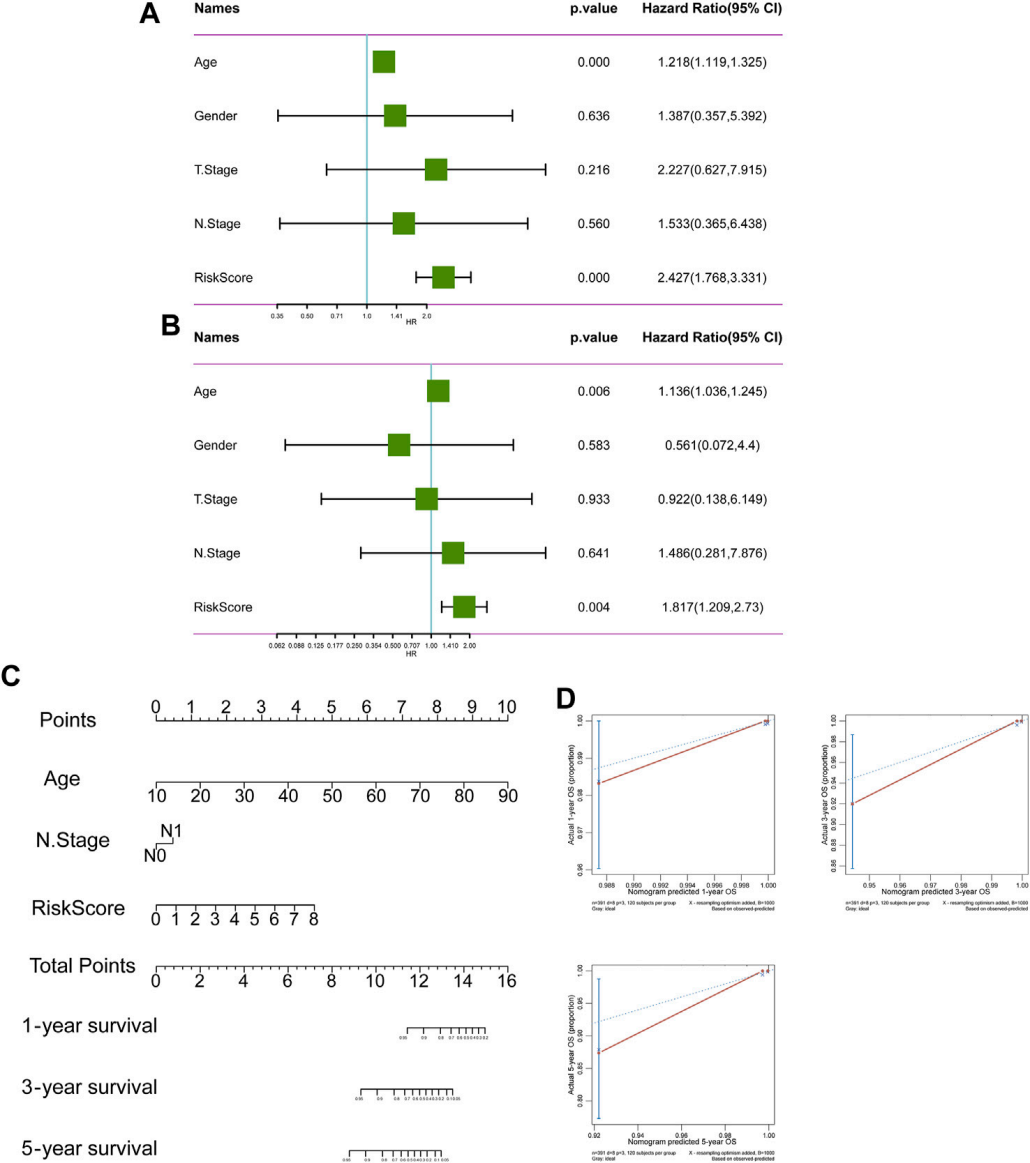
The prognostic value of risk score. **(A, B)** The forest plot of OS univariate and multivariate COX model analysis. **(C)** The nomogram plot predicting the overall survival probability by age, N stage and risk score. **(D)** The Calibration curves of the nomogram using the bootstrap method in internal validation.

526 DEGs were generated from differential gene analysis. Combined with TCGA-THCA survival data, 46 prognostic differentially expressed target genes were determined. To prevent overfitting, LASSO Cox regression was used to establish a more precise prognostic model (Figure 4A). After LASSO analysis, 4 hub genes were identified as a prognostic signature, including SNAI1, STC1, PKHD1L1 and ANKRD37. The risk score was generated as the following equation: RS= (0.04*SNAI1exp.) + (0.016*STC1exp.) + (0.008*PKHD1L1exp.) + (0.099*ANKRD37exp.) (Figure 4B). To establish and validate the prognostic value of the risk score, TCGA-THCA database was divided into a training dataset and a validation dataset through machine learning algorithm by caret R package. In each dataset, patients were divided into high-risk group and low-risk group, the Kaplan–Meier survival analysis demonstrated a significant overall survival difference between two groups both in training and validation dataset (*p* < 0.05) (Figures 4C,D). The ROC curve of this signature is shown and the AUC values for 1, 3 and 5 years is calculated (Figures 4E,F). We further identified age and the risk score as an independent prognostic factor by univariate and multivariate Cox regression analysis (Figures 5A,B). Using clinical data from TCGA dataset, we created a nomogram based on the risk score, N stage and age (Figure 5C). The 1-, 3-, and 5-year calibration curves were further implemented to show the nomogram model performed well on the robustness and efficacy (Figure 5D).

### 3.5 Functional analysis and genomic features beneath the risk score

**FIGURE 6:**
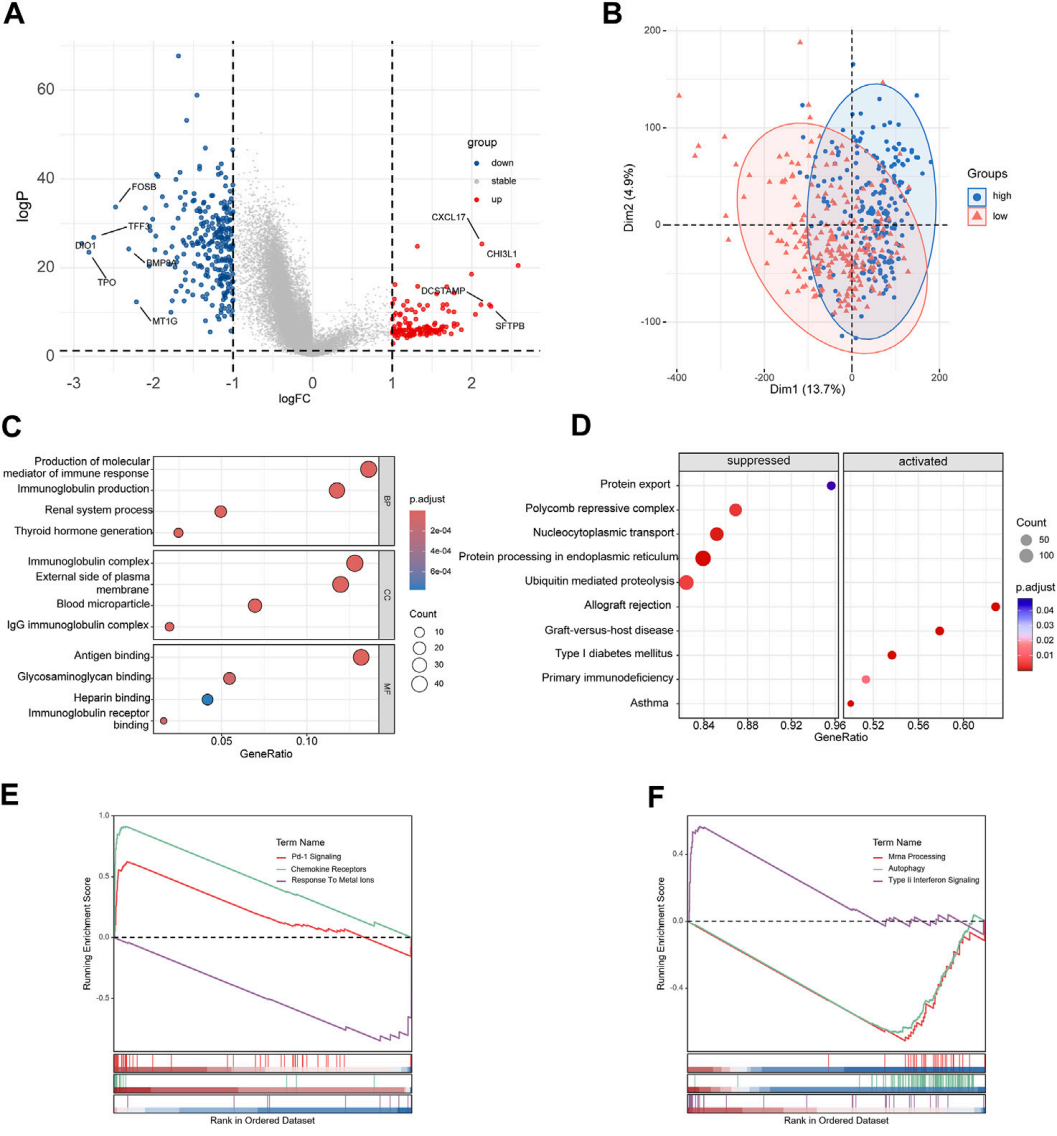
Functional enrichment analyses of differentially expressed genes. **(A)** Volcano plot of DEGs between high-risk group and low-risk group. **(B)** Principal component analysis of DEGs. **(C)** Results of GO enrichment analysis. **(D)** Results of KEGG pathway enrichment analysis. **(E, F)** Results of Reactome pathway and WikiPathways enrichment analysis.

**FIGURE 7:**
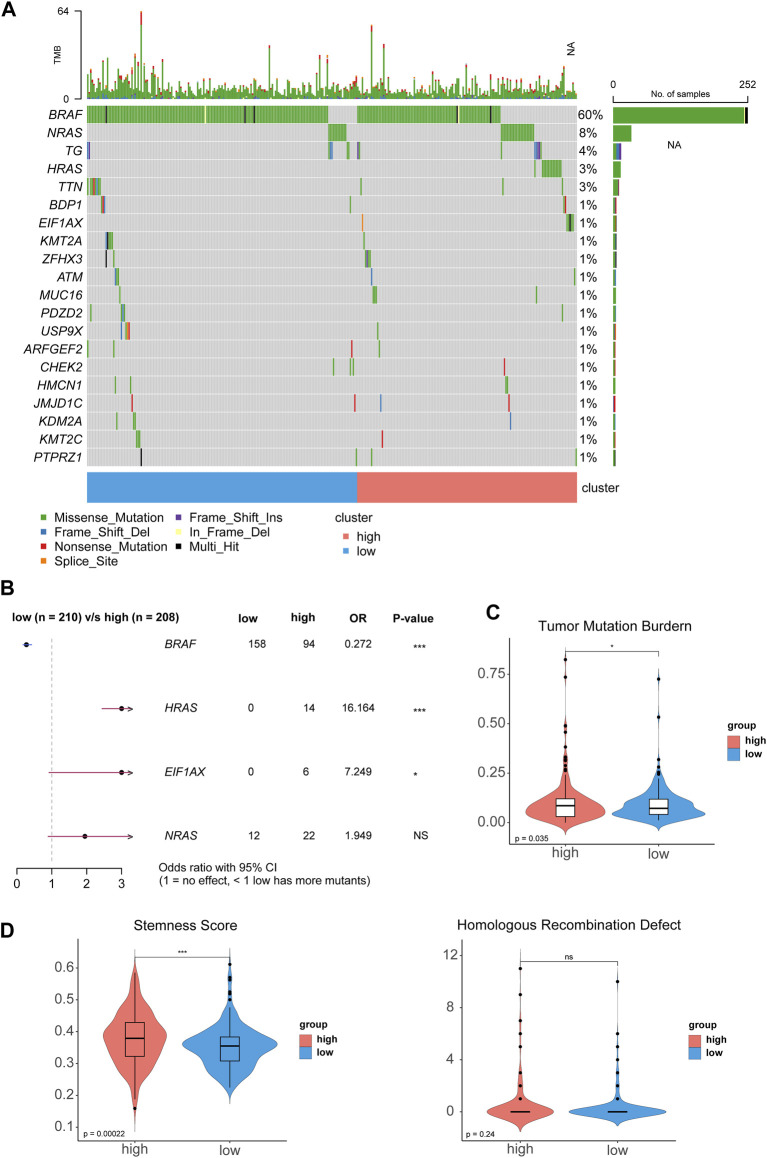
The genomic analysis of high-risk group and low-risk group. **(A)** The waterfall plot displays the somatic and methylated landscape between two groups. **(B)** The forest plot of differentially mutated diver genes of thyroid cancer. **(C, D)** The violin plot of tumor mutation burden, stemness score and homologous recombination defect.

The DEGs between high-risk group and low-risk group were displayed in the volcano plot and principal component analysis, in which 130 genes were upregulated and 255 were downregulated (Figures 6A, B). Next we performed functional enrichment analysis of these 385 DEGs. GO analysis showed that DEGs mainly enriched in ‘production of molecular mediator of immune response’ in BP, ‘immunoglobulin complex’ in CC and ‘antigen binding’ in MF (Figure 6C). The result of KEGG pathway analysis showed ‘Allograft rejection’ was the most activated pathway and ‘Protein export’ was most suppressed pathway (Figure 6D), and detailed correlation analysis between KEGG pathway and risk score was also performed (Supplementary Figure S2). With further investigation in pathway enrichment, ‘Pd-1 signaling’ and ‘chemokine receptors bind chemokines’ were upregulated in Reactome pathway and ‘Type II Interferon Signaling’ was upregulated WikiPathways (Figures 6E, F). The analysis of genomic features was also conducted to evaluate the characteristic of tumor microenvironment. The gene mutation pattern of high-risk group and low-risk group was shown in Figure 7A. The differentially mutated diver genes between high-risk group and low-risk group were illustrated in a forest plot, including BRAF, HRAS, EIF1AX and NRAS (Figure 7B). The high-risk group was significantly enriched in the mutation of HRAS and EIF1AX. In accordance with the results above, the high-risk group hold a higher tumor mutation burden (Figure 7C). In addition, high-risk group showed higher stemness scores but no difference in homologous recombination defect estimation (Figure 7D). Based on these genomic features, the therapeutic evaluation of targeted therapy in advanced thyroid cancer showed that the Motesanib, Lapatinib and Sunitinib might exert a possible therapeutic effect in the high-risk group (Supplementary Figure S3) (Fu et al., 2023). These results suggested that the risk score model had a strong connection to tumor immune microenvironment in PTC and affects the genomic status and mutation load.

### 3.6 The immune landscape of high-risk groups in PTC

**FIGURE 8:**
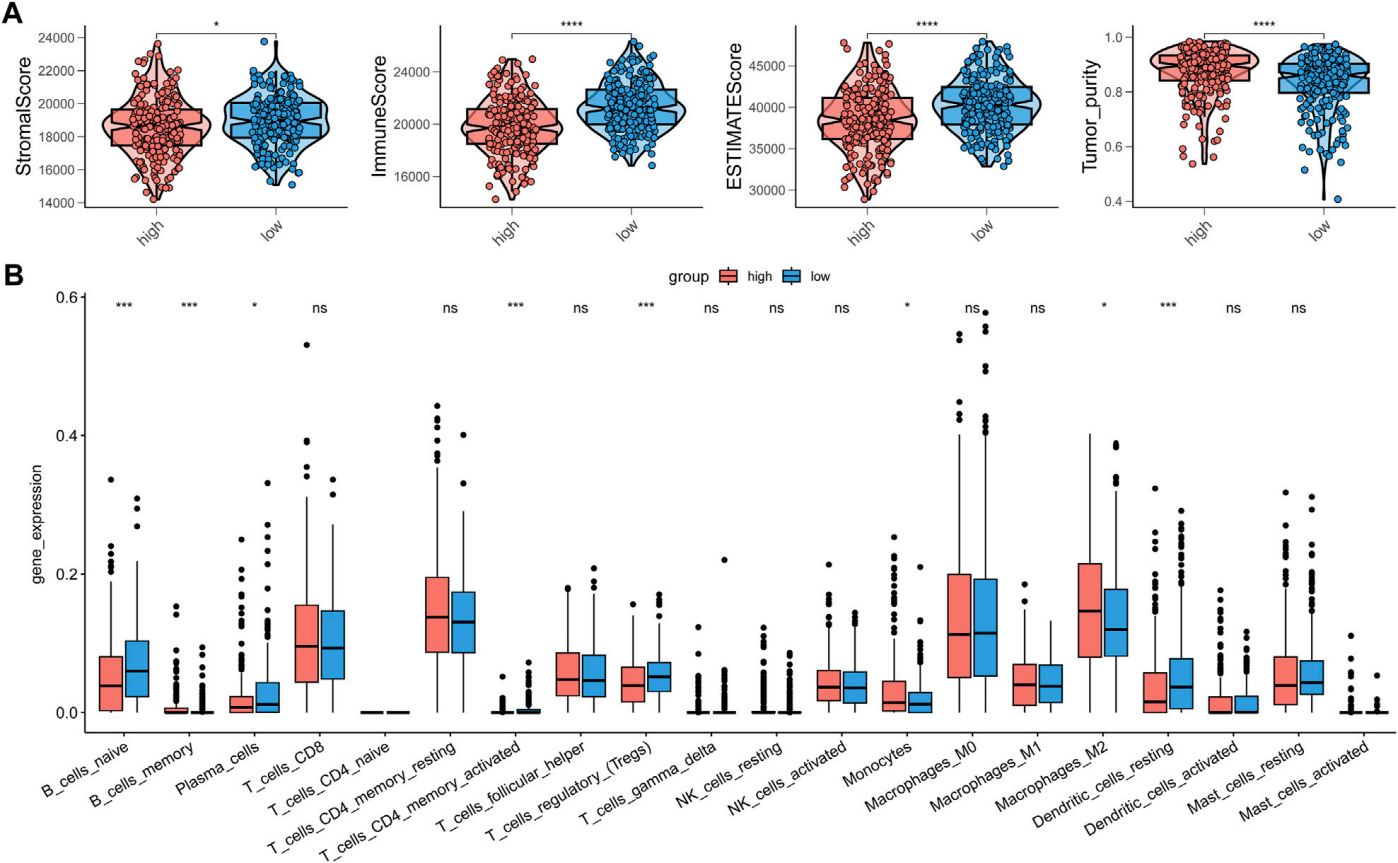
The correction between immune infiltration and risk score. **(A)** The estimation of stromal score, immune score and tumor purity by ESTIMATE. **(B)** The detailed immune cells infiltration analysis by CIBERSORT between high-risk group and low-risk group.

**FIGURE 9:**
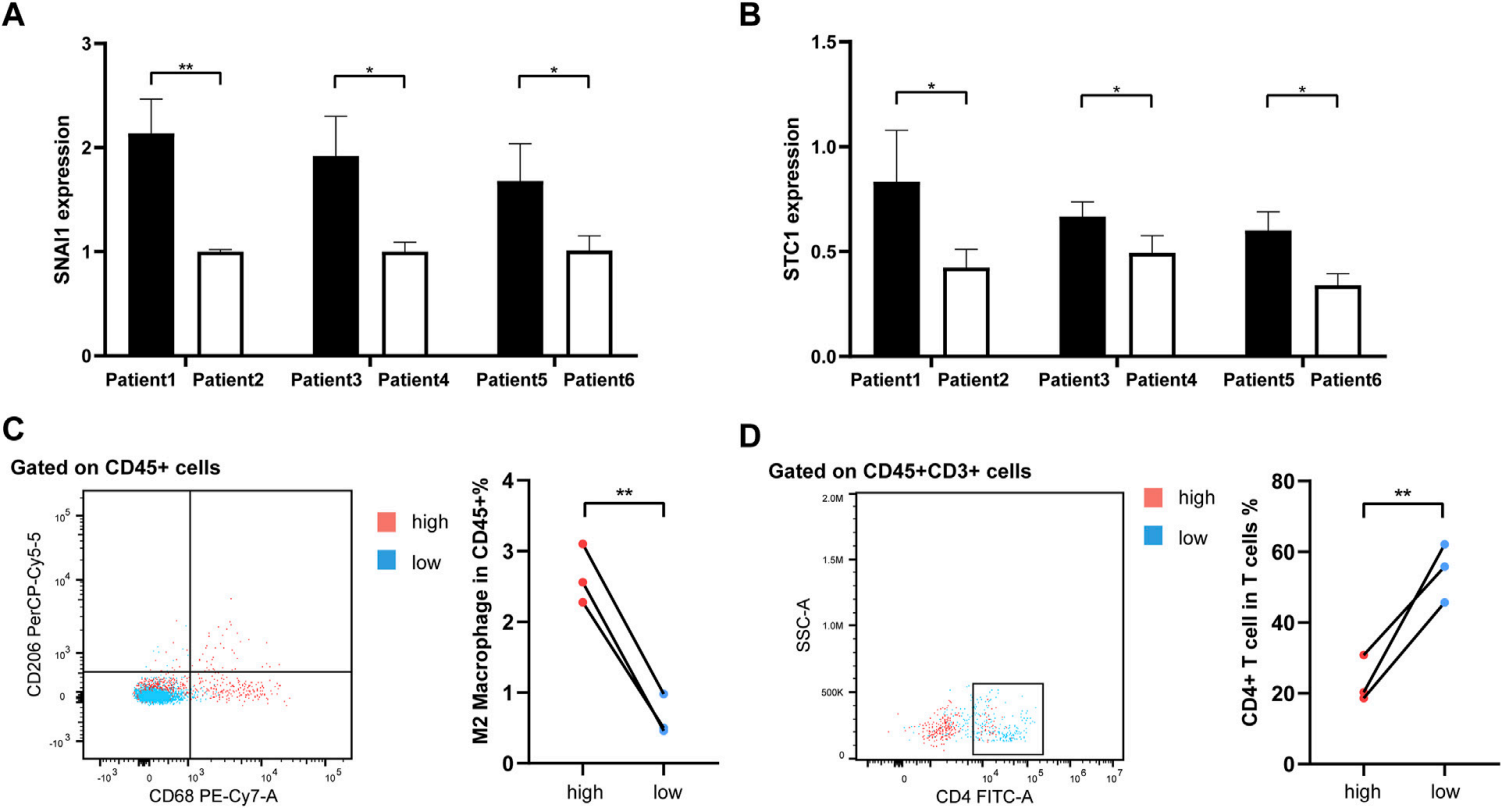
The flow cytometry validation of immune infiltration. **(A, B)** The histograms representing qRT-PCR of hub genes SNAI1 and STC1 in 6 PTC samples. **(C)** The infiltration of M2 macrophages (CD45^+^CD68^+^CD206^+^) between 2 groups. **(D)** The infiltration of CD4^+^ T cells (CD45^+^CD3^+^CD4^+^) between 2 groups.

As our understanding of the tumor immune microenvironment deepens, the level of immune cell infiltration is thought to be closely related to tumor progression. With a microenvironment rich in immune cells, it is believed that immune system plays a key role in cancer prevention as well as in its initiation and progression in thyroid cancer (Menicali et al., 2020). The connection between our risk score and infiltration of immune cells was explored. The stromal and immune score and tumor purity was computed by ESTIMATE algorithm (Figure 8A). It was shown that in the high-risk group the samples were infiltrated by higher tumor cells and lower stromal cells and immune entities. The more detailed immune cell infiltration evaluation was done by CIBERSORT. We found a higher infiltration level of memory B cells, monocytes and macrophages especially M2 type macrophages in the high-risk group. The risk score was also negatively correlated with the abundance of naïve B cells, activated CD4^+^ T cells, Treg cells and resting dendritic cells (Figure 8B). To further validate database based immune infiltration evaluation, we picked 3 pairs of PTC patients with a different expression of the hub genes of the risk score, SNAI1 and STC1 (Figures 9A, B). Six samples were divided into two groups with the expression level of the hub genes. Patient sample 1, 3, 5 in high group with higher expression of SNAI1 and STC1, while patient sample 2, 4, 6 in low group. We measured the infiltration level of M2 macrophages (CD45^+^CD68^+^CD206^+^) and CD4^+^ T cells (CD45^+^CD3^+^CD4^+^) by flow cytometry between two groups. In accordance with the estimated results, the samples with a high expression of the hub genes showed a higher infiltration of M2 macrophages and lower level of CD4^+^ T cells (Figures 9C, D). It is suggested that the risk score is correlated with an immuno-suppressive microenvironment in PTC.

## 4 Discussion

In our study, we constructed a novel 4-gene risk model associated with disulfidptosis based on a combination of the DRGs and scRNA sequencing data of papillary thyroid carcinoma. As a newly discovered type of programmed cell death, disulfidptosis is considered to be closely related to the occurrence and development of tumors as ferroptosis and cuproptosis death which were fully explored in the past (Feng et al., 2023; Hadian and Stockwell, 2023; Zhang et al., 2023). In-depth exploration of these cell death processes will help us to deepen our understanding of the mechanisms behind tumor development and provide evidence for investigating new treatments. Compared with other solid tumors, the exploration of the mechanisms behind thyroid tumors and its microenvironment is still in the initial stage, and the understanding of tumor ecosystem is also relatively lacking (Yin et al., 2020). With the application of scRNA seq techniques in thyroid cancer, we have gained a deeper understanding of the tumor microenvironment at the cellular level (Pu et al., 2021). Previous studies have demonstrated that signatures generated from scRNA transcriptome could estimate cell type abundance in bulk transcriptome (Newman et al., 2019; Zhao et al., 2024). The signatures based on disulfidptosis were reported closely related to clinical prognosis and immune microenvironment characteristics in hepatocellular cancer (Wang et al., 2023), bladder cancer (Zhao et al., 2023), lung adenocarcinoma (Qi et al., 2023) and breast cancer (Xia et al., 2023). Therefore, this study combines disulfidptosis related genes with scRNA sequencing data of thyroid papillary carcinoma, hoping to fertilize the understanding of thyroid cancer exploration.

By scoring DRGs evaluation in various cell components of thyroid cancer, we found that endothelial cells had the best association with disulfidptosis. Endothelial cells are mainly involved in abnormal angiogenesis in tumor development, and they are thought to be closely related to changes in tumor metabolic pathways during recruitment (Harjes et al., 2012). Disulfide accumulation due to abnormal glucose metabolism is the cause of disulfidptosis, so we indicate that disulfidptosis related endothelial cells is an important part of the special metabolic environment in thyroid tumor. Our prognostic model was based on this result. Meanwhile, endothelial cell metabolism is an emerging target for anti-angiogenic therapy in tumor angiogenesis and choroidal neovascularization (Rohlenova et al., 2020). Since the application of anti-angiogenic drug Lenvatinib in refractory advanced thyroid cancer has been well reported (Schlumberger et al., 2015), the risk model assessment of disulfidptosis related endothelial cells in thyroid cancer may provide potentially sensitive patient profiles for the therapy.

We established the prognostic model by LASSO Cox regression, and finally obtained the risk score based on SNAI1, STC1, PKHD1L1 and ANKRD37 genes. According to further experimental validation in clinical samples, SNAI1 and STC1 showed significant differences in expression among different patients. SNAI1, snail family transcriptional repressor 1, it is involved in regulating EMT processes in pancreatic tumor cells (Zhang et al., 2022), SNAI1 is thought to induce tumor stemness and resistance to radiation in colon cancer (Zhu et al., 2018) and unstable expression of SNAI1 leads to distant metastasis in lung cancer (Wang et al., 2019). In pan-cancer studies, high expression of SNAI1 is mainly involved in the enhancement of stemness and migration of tumor cells, which significantly increases the malignant biological manifestation of tumors. STC1, Stanniocalcin 1, it has been found to promote metastasis, lipid metabolism and cisplatin chemoresistance in ovarian cancer (Lin et al., 2022), STC1 is mainly involved in STAT3-mediated proliferation in breast cancer (Avalle et al., 2022) and STC1 expression is thought to play an important role in driving tumor immune resistance (Lin et al., 2021). SNAI1 and STC1 contribute to accelerating tumor progression in tumor growth, chemotherapy resistance, and immune environment suppression. While these two genes are still less studied in thyroid cancer (Hardy et al., 2007; Hayase et al., 2015), our study suggests that exploring their function in thyroid cancer is a potential direction for advancing thyroid cancer therapy.

Our study also explored the detailed characteristics of the risk scores, which we found to be strongly associated with the immune microenvironment of thyroid cancer. Although the infiltration of immune cells was reduced in the high-risk group, the infiltration of tumor-promoting immune components such as M2 macrophages was significantly increased, consistent with previous findings on the immune microenvironment of advanced thyroid cancer (Menicali et al., 2020). Immunotherapy may offer a new hope in patients with advanced thyroid cancer due to resistance to RAI therapy, inapplicability of chemotherapy drugs and poor response to targeted drugs (Haddad et al., 2018). However, the exploration of the immune microenvironment of thyroid cancer still remains a long way off.

## Data Availability

The original contributions presented in the study are included in the article/Supplementary Material, further inquiries can be directed to the corresponding authors.
