# Supplementary figures and images for: A novel prognostic signature and immune microenvironment characteristics associated with disulfidptosis in papillary thyroid carcinoma based on single-cell RNA sequencing
Source: Front Cell Dev Biol. 2023 Nov 14;11:1308352. doi: 10.3389/fcell.2023.1308352 (PMC10682199; doi:10.3389/fcell.2023.1308352)

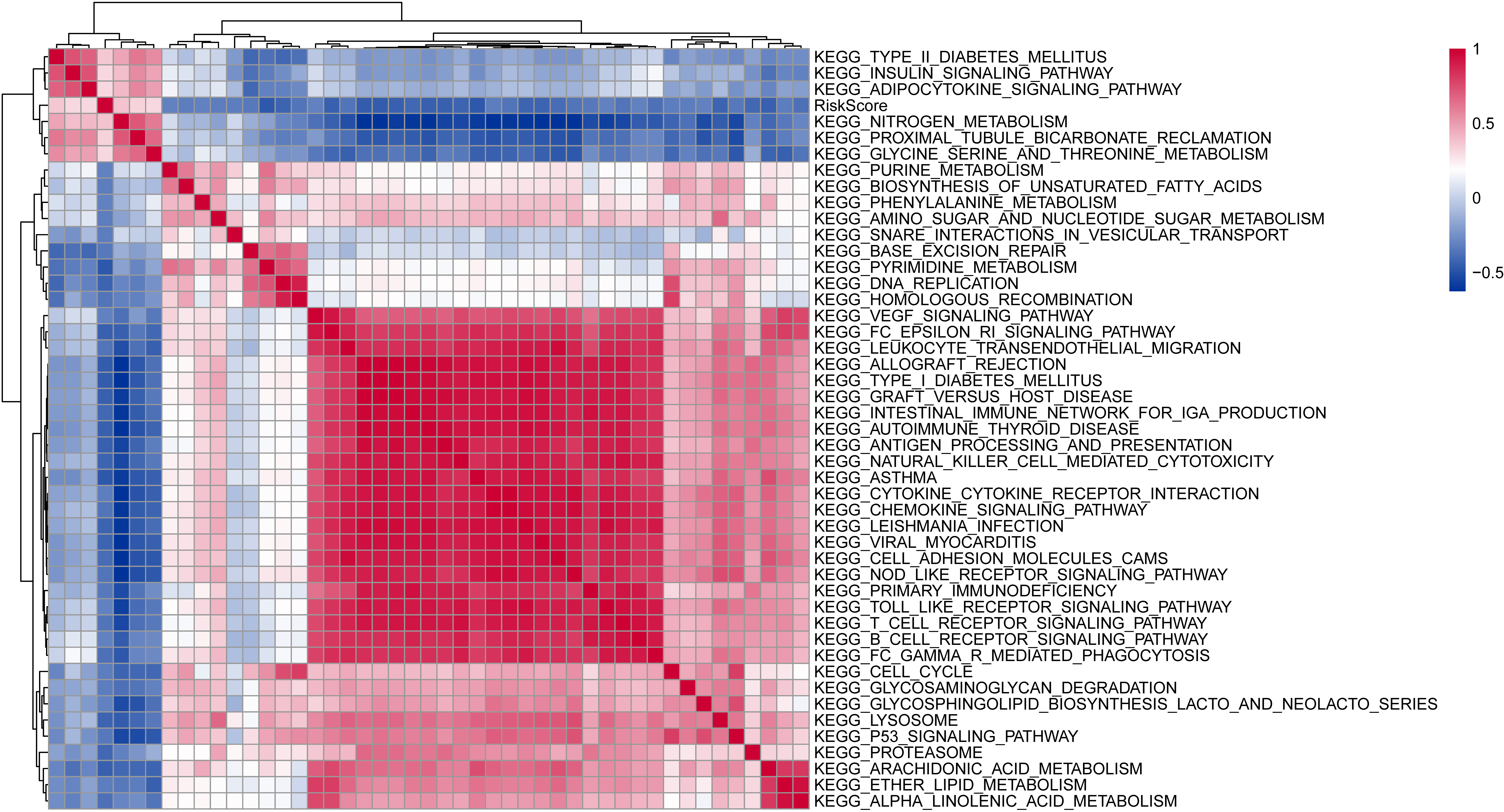

Supplement: Supplementary file 2 [file Image2.TIF]

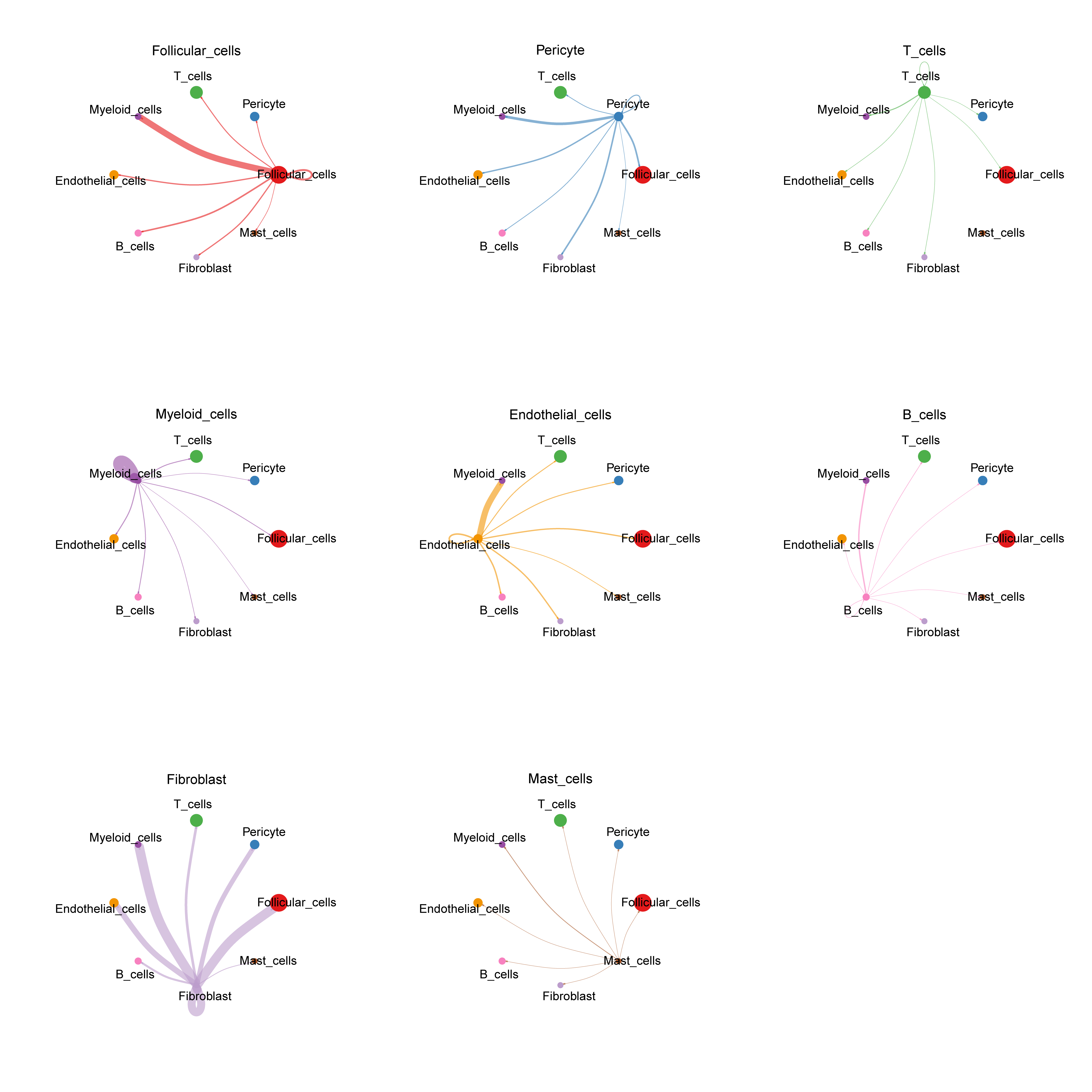

Supplement: Supplementary file 3 [file Image1.TIF]
